# Developing Up-Scale Allogeneic Chondrocyte Therapies Using Juvenile Donor Cartilage

**DOI:** 10.3390/ijms26199566

**Published:** 2025-09-30

**Authors:** Charlotte H. Hulme, Jade Perry, Helen S. McCarthy, Tian Lan, Thavisha Ranasinghe, Nigel Kiely, Robert Freeman, Jonathan Wright, Karina T. Wright

**Affiliations:** 1 Centre for Regenerative Medicine Research, School of Life Sciences, Keele University, Keele, Stoke-on-Trent ST5 5BG, Staffordshire, UK; charlotte.hulme@nhs.net (C.H.H.); jade.perry3@nhs.net (J.P.); helen.mccarthy6@nhs.net (H.S.M.); tian.lan@nhs.net (T.L.); 2 Oswestry Keele Orthopaedic Research Group, Robert Jones and Agnes Hunt Orthopaedic Hospital Foundation Trust, Oswestry SY10 7AG, Shropshire, UK; thavisha.ranasinghe@nhs.net (T.R.); nigel.kiely@nhs.net (N.K.); robertfreeman@nhs.net (R.F.); 3 Limb Reconstruction Unit, Royal National Orthopaedic Hospital, Stanmore, London HA7 4LP, UK; 4 National Institute for Health and Care Research (NIHR) Birmingham Biomedical Research Centre, Birmingham B15 2GW, West Midlands, UK

**Keywords:** chondrocytes, hollow-fibre bioreactor, large-scale expansion, allogeneic cell therapy, cartilage cell repair, polydactyly chondrocytes, iliac apophysis chondrocytes

## Abstract

Allogeneic chondrocyte therapies present an attractive alternative to existing autologous therapies for the repair of cartilage defects, enabling the selection of optimal donor cells and streamlined manufacturing processes. This study investigates the potential of juvenile chondrocytes derived from human infantile (aged 0–4 y) polydactyly digits and the iliac apophysis for cartilage repair using Good Manufacturing Practice bioreactor expansion. Iliac apophysis (*n* = 4) and polydactyly tissues (*n* = 4) were assessed histologically. Chondrocytes were isolated enzymatically and cultured using standard tissue culture plastic (TCP) methodology. Upon sufficient cell expansion, chondrocytes were seeded into the Quantum^®^ bioreactor system or onto TCP (±vitronectin coating). The manufactured chondrocytes growth rates, total cell yields, chondrogenic pellet forming capacity (GAG/DNA, histology), immunoprofiles (flow cytometry) and gene expression (RT-qPCR) were assessed. Equivalent chondrocyte numbers were isolated from polydactyly and iliac apophysis donors per wet weight of tissue. Quantum^®^-expanded chondrocytes from both sources yielded comparable cell numbers; however, growth was slowed in the Quantum^®^ compared to TCP. Polydactyly and iliac apophysis-derived chondrocytes expressed chondrocyte cell surface markers (CD166, CD44, CD151, SOX9) and formed chondrogenic pellets. Quantum^®^ bioreactor expansion did not alter, gene expression or capacity to form glycosaminoglycans (GAGs (normalised to DNA content)) compared to matched TCP expansion. Juvenile cartilage donors are a promising chondrocyte source for the development of an allogeneic therapy. This novel study expanding juvenile chondrocytes in the Quantum^®^ GMP-compliant bioreactor suggests that culture conditions may need modification to improve growth, whilst retaining cartilage forming capacity.

## 1. Introduction

Defects of the articular cartilage commonly lead to the development of osteoarthritis (OA) if left untreated. The exact prevalence of cartilage defects is not defined, as patients only typically present clinically if they are symptomatic, e.g., in pain or with reduced joint function [1]. However, cartilage lesions have been reported in about 66% of knee arthroscopies [2,3]. Multiple approaches for cartilage repair exist, several of which utilise cell-based therapies with the aim of regenerating/repairing cartilage [4].

To date, cartilage repair using Autologous Chondrocyte Implantation (ACI) or matrix-associated ACI (MACI) is generally successful, especially when long-term outcomes are considered [5,6,7]. However, there are limitations of ACI, including difficulties with the logistics of manufacturing individual patient’s cells and the requirement of specialist surgical expertise to deliver the therapy [8]. Furthermore, there is additional burden to patients having to undergo two surgical procedures: the first to harvest healthy cartilage and the second to implant the culture-expanded cells [9]. In order to offer an advanced therapy medicinal product (ATMP) to repair cartilage defect(s) to a large number of patients, allogeneic cellular therapies need to be developed [4,10]. The use of chondrocytes in allogeneic cartilage repair strategies provides a non-immunogenic choice [11], compared with other stromal cell alternatives.

A move towards manufacture of allogeneic chondrocyte therapies will offer the opportunity to select optimal populations of chondrocytes, both in terms of specific donor characteristics and donor tissue source. Moreover, optimal manufacturing protocols can be developed to ensure large numbers of chondrocytes can be grown in a streamlined, reproducible process. We have previously demonstrated that adult chondrocytes, from osteoarthritic joints (knee arthroplasty), can be up-scale manufactured using a Good Manufacturing Practice (GMP)-compliant hollow fibre bioreactor (Quantum^®^, TerumoBCT) [12]. The use of a bioreactor to manufacture large numbers of chondrocytes would be more cost-effective to produce multiple cell ‘doses’ for the development of an ‘off-the-shelf’ therapy. The Quantum^®^ bioreactor system has been widely demonstrated to support this cost-effective manufacturing process [13].

Optimal chondrocyte donor sources now need to be identified that are likely to be more potent in repairing cartilage than that of adult arthroplasty donors. Chondrocytes derived from juvenile cartilage tissues are thought to retain greater differentiation potential; the skeletally immature donor cartilage still retaining a thicker articulating layer and with no calcified zone. Juvenile hyaline cartilage from synovial joints, e.g., the knee/ankle can be harvested from cadaveric child donors; however, this donor supply is limited, particularly in the limited time window where cartilage remains immature. Particulated allogeneic juvenile cartilage (from donors aged 13 years and under) has been used clinically to repair articular cartilage for over 15 years in at least 8700 cases (commercialised as DeNovo^®^ NT Natural Tissue Graft from Zimmer) [14], with outcomes being promising in terms of knee pain and improved function at two years post-treatment [15]. However, it is believed that cartilage obtained from infantile donors retains the greatest regenerative capacity (aside from foetal tissues). Ethical access to foetal tissues is, however, limited.

In this study, we have assessed two sources of juvenile chondrocytes, which can be accessed through routine orthopaedic surgeries. The first is from infants/young children (0–4 years) undergoing surgery to remove extra digits for the treatment of polydactyly. The second is from the iliac apophysis (IA; hip growth plate) of infants (0–3 years), harvested during surgery to treat hip dysplasia.

Polydactyly is a congenital malformation in which infants are born with extra fingers and/or toes [16]. Polydactyly digits (PD) could serve as a reliable source of allogeneic tissue; polydactyly occurring in 1 in 3000 births with supernumerary little fingers and toes (postaxial side of hands or feet) and 1 in 1000 births with extra thumbs (preaxial side of hand) [17]. Genetic engineered PD-derived chondrocytes, retrovirally transduced to express Transforming Growth Factor (TGF)-β1, in a 3:1 ratio with non-transformed PD chondrocytes are being used for the treatment of OA in South Korea (INVOSSA; Tonogenchoncel-L, TissueGene-C, produced by KOLON TissueGene, Rockville, MD, USA) and phase III trials resumed in the United States in 2021. These cells have been demonstrated as safe in preclinical models [18]; safety (and efficacy) were later confirmed in human trials for the treatment of OA patients with chronic, grade III disease [19,20]. Non-engineered PD chondrocytes proliferated more rapidly and demonstrated a lower level of de-differentiation (1 × 10^3^-fold decrease vs. 1 × 10^5^-fold in Collagen 2 expression) compared to adult chondrocytes [17]. Additionally, they can be maintained in sheets for cartilage repair [21], producing lower levels of the matrix degrading enzyme, matrix metalloproteinase-3, compared to adult chondrocyte sheets [22] and have been implanted into 10 patients as a treatment for moderate to severe OA (Kellgren Lawrence grade 2–4) [23]. Histological analysis of the regenerated tissue biopsies at 12 months post transplantation demonstrated high proteoglycan content (assessed via Safranin-O staining) and high-quality repair tissue (average International Cartilage Repair Society (ICRS) score of 80.8; where 0 represents fibrous tissue and 100 = hyaline cartilage)) but with a mix of hyaline and fibrocartilage (staining for both types I and II collagen) [23].

The potential of juvenile hip chondrocytes has been less widely described. Kreuz et al. (2013) indicated that hip cartilage taken from children aged 1–10 with hip dysplasia or Perthes disease has a high proteoglycan content, with the superficial layer expressing type II collagen and the remainder of the tissue being positive for type I collagen [24]. Chondrocyte cells with a fibroblast-like morphology could be extracted and expanded in monolayer and in a 3D PGA-fibrin scaffold, forming ECM between the cells, with no expression of types II or X collagen and induction of cartilage marker genes after three weeks in culture (type II collagen, type IX collagen and Cartilage Oligomeric Protein (COMP) [24]. However, the number of published studies that have utilised this cartilage donor source for tissue regenerative strategies is limited and additional characterisation of the cell products is required to determine their potential for future cartilage repair therapies.

The development of GMP protocols to up-scale manufacture juvenile chondrocytes have the potential for novel cell-based therapies to treat large numbers of patients, as well as large chondral defects in a cost-effective manner. This study aimed to determine whether chondrocytes from two juvenile donor sources, PD and the IA, could be extracted and up-scale culture expanded in the Quantum^®^ bioreactor. Further, the chondrogenic potential of the cells following bioreactor manufacture or using standard 2D tissue culture plastic was assessed.

## 2. Results

### 2.1. Participants

Samples were collected from four polydactyly patients undergoing surgery to remove additional digits. IA was collected from four patients during open reduction or Salter’s osteotomy surgeries. The individual donors were all aged 0–4 (Table 1).

### 2.2. Histological Analysis of Native Infant Cartilage

**Figure 1:**
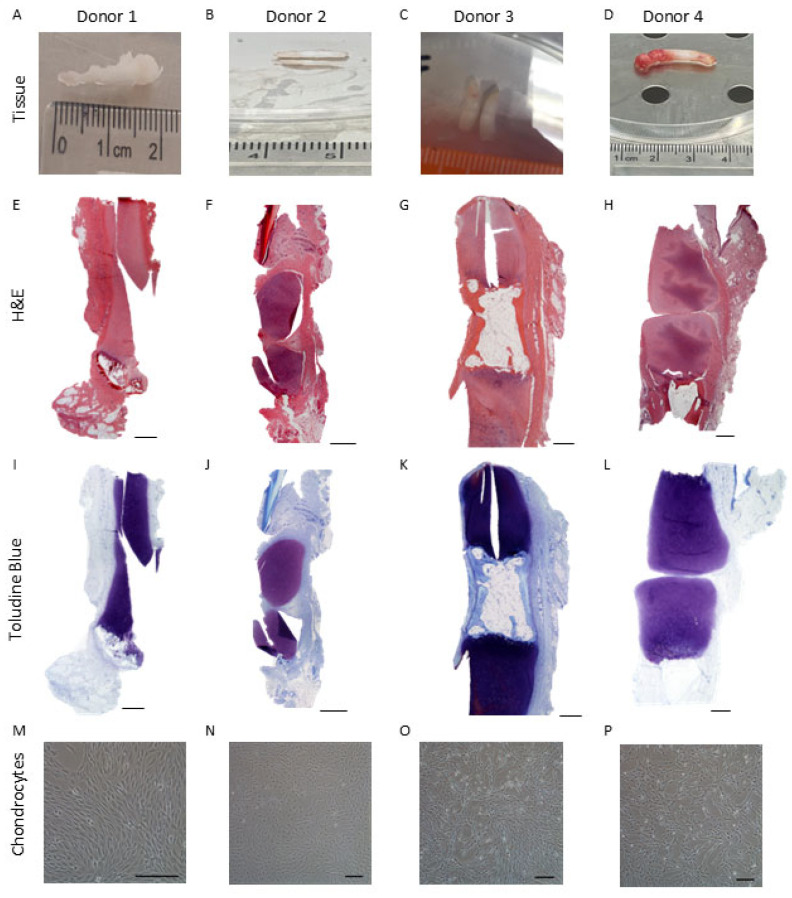
Isolation of chondrocytes from Polydactyly digit cartilage. (**A**–**D**) Cartilaginous like tissue was dissected from four donors. Histological analysis of the isolated cartilage was performed using (**E**–**H**) H&E and (**I**–**L**) toluidine blue to assess cartilage proteoglycan content. (**M**–**P**) Representative images of the cells isolated from the different polydactyly cartilage donors. Scale bars represent 500 µm.

**Figure 2:**
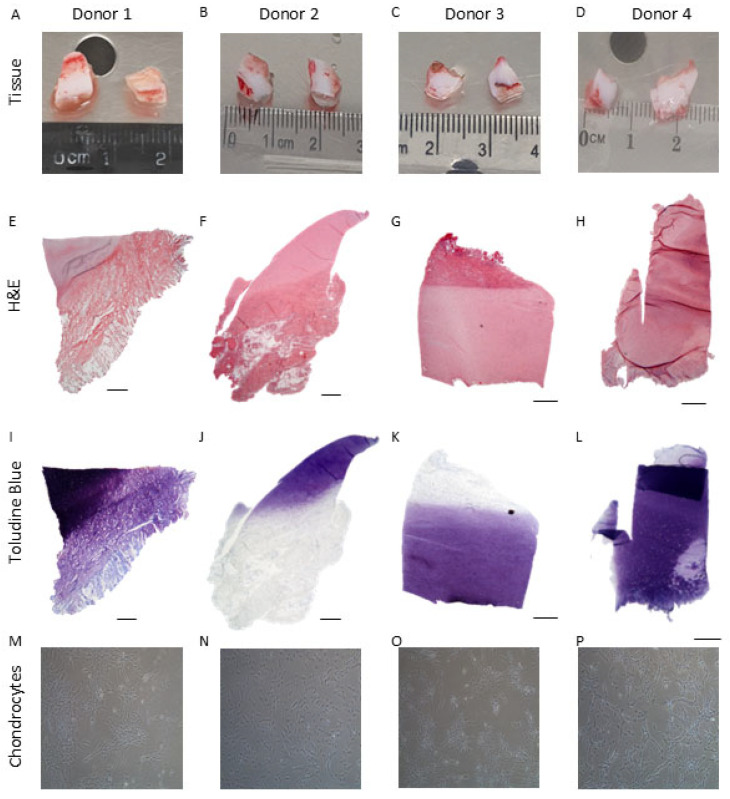
Isolation of chondrocytes from iliac apophysis tissue. (**A**–**D**) Iliac apophysis samples were collected with informed consent from four donors aged 0–4. Histological analysis of the isolated cartilage was performed using (**E**–**H**) H&E and (**I**–**L**) toluidine blue to assess cartilage proteoglycan content (**M**–**P**) Representative images of the cells isolated from the different polydactyly cartilage donors. Scale bars represent 500 µm.

Under polarised light, both PD and IA samples demonstrated a hyaline cartilage appearance. The PD samples were well populated with cells of a rounded morphology, whereas cells within the IA samples were smaller and more spindle shaped. PD samples demonstrated an obvious orientation, with more developmentally mature features present in some samples such as parallel collagen fibres and aligned chondrocytes indicating an “apparent” articulating surface with hypertrophic chondrocytes and the formation of a tidemark on the opposing side of the biopsy. IA samples were less orientated; no bone was present, but some hypertrophic chondrocytes could be seen. Both PD and IA samples demonstrated good to excellent matrix metachromasia (Figure 1 and Figure 2).

### 2.3. Chondrocyte Growth and Cell Morphology

Chondrocytes could be isolated from PD and IA tissues, with both tissue types yielding equivalent cell numbers per wet weight of tissue (PD: 6.12 ± 3.16 × 10^3^ cells/mg; IA: 5.83 ± 4.4 × 10^3^ cells/mg; *p* > 0.05; Mann–Whitney test). Detailed data for yield and growth kinetics is included in Table 2 and Table 3. PD chondrocytes retained similar morphologies across donors, typically retaining a fibroblastic-like morphology across passages. In general, IA-derived chondrocytes had a more typical ‘chondrocyte’-like rounded/polygonal shape upon isolation and typically grew out from colonies, demonstrating a more fibroblastic morphology with passage (Figure 1 and Figure 2). Once isolated and culture expanded on TCP to achieve sufficient cell numbers (10 × 10^6^) to seed the bioreactor, chondrocytes from both donor tissue types could be maintained and culture expanded in the Quantum^®^ bioreactor.

**Figure 3:**
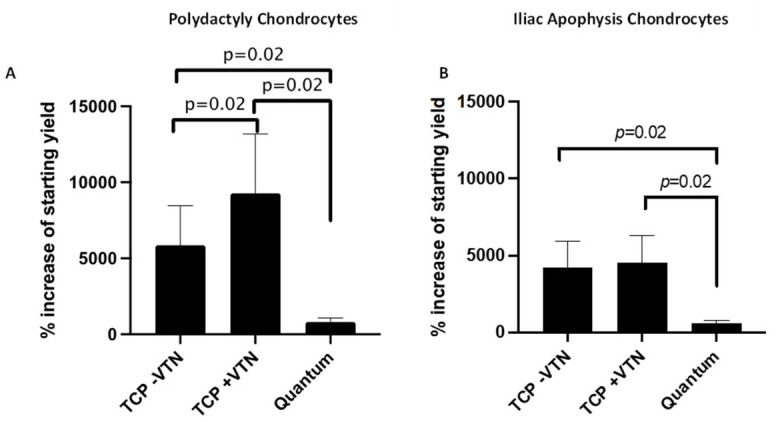
Cell yields of polydactyly and iliac apophysis chondrocytes following tissue culture plastic (TCP) or Quantum^®^ expansion. The % increase in the number of cells harvested compared with the number seeded following expansion in the Quantum^®^ bioreactor or TCP coated/uncoated with vitronectin for (**A**) polydactyly-derived and (**B**) iliac apophysis-derived chondrocytes. (**A**,**B**) TCP expansion with and without vitronectin coating resulted in an increased % starting yield compared to Quantum^®^ expansion for chondrocytes from both donor sources (paired *t*-test; data are mean ± SD).

**Figure 4:**
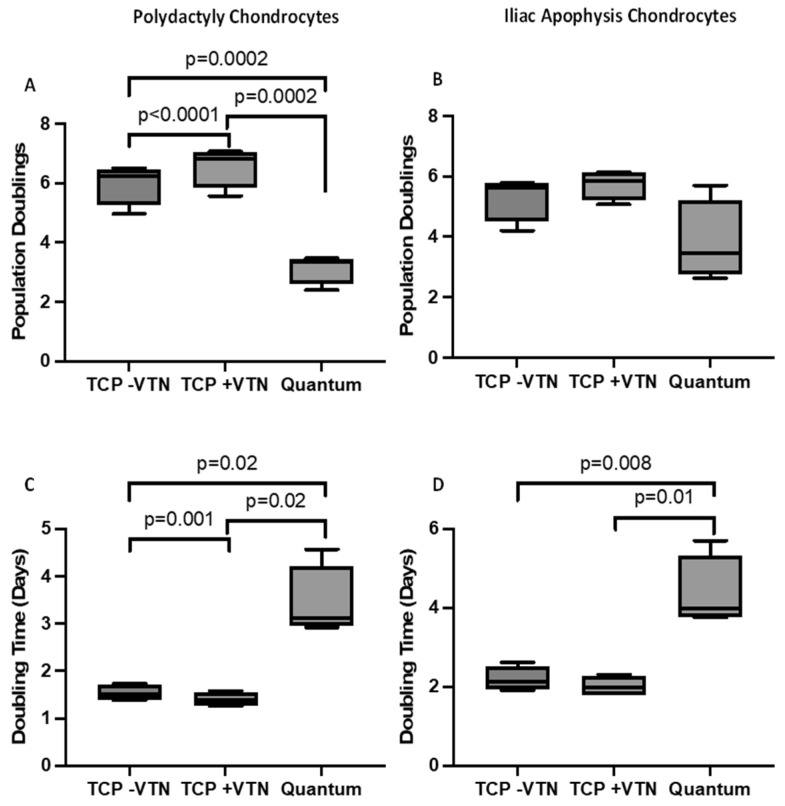
Growth kinetics of chondrocytes expanded in the Quantum^®^ bioreactor or on tissue culture plastic (TCP) coated or uncoated with vitronectin. (**A**) Polydactyly-derived chondrocytes had increased population doublings on TCP compared to when manufactured in the Quantum^®^ (paired *t*-test). Vitronectin coating TCP also increased the number of population doublings. (**B**) Expansion on TCP or in the Quantum^®^ did not alter the number of population doublings for iliac apophysis chondrocytes. (**C**) The doubling time of polydactyly chondrocytes was higher following Quantum^®^ manufacture compared with TCP, with vitronectin-coated TCP demonstrating the lowest doubling time (paired *t*-test). (**D**) Iliac apophysis chondrocytes had lower doubling times when manufactured on TCP cf. Quantum^®^. Data are mean ± SD.

Following Quantum^®^ expansion, 92 ± 27 × 10^6^ PD-derived chondrocytes could be yielded from an initial seeding of 10 × 10^6^ following 10.5 ± 0.6 days (data are mean ± SD; Table 2). From all eight Quantum^®^ expansions, it was a PD chondrocyte donor that provided the highest overall yield of 110.8 × 10^6^ cells following 11 days in culture (Table 2). Notably, the range of Quantum^®^ harvest yields was quite varied from the PD chondrocytes, with one expansion only yielding 53 × 10^6^ cells following 11 days in culture (Table 2). Despite chondrocyte expansion in the GMP-compliant closed system being possible, the % increased yield of the starting cell number was significantly lower compared to when expanded on either uncoated or vitronectin-coated TCP (Figure 3). Coating TCP with vitronectin resulted in an increased % cell yield compared to uncoated TCP (*p* = 0.02; paired *t*-test; Figure 3A). Furthermore, Quantum^®^ expansion resulted in fewer population doublings (Figure 4A) and an increased doubling time (Figure 4C) compared to TCP expansion. PD chondrocytes expanded on vitronectin-coated TCP demonstrated increased population doublings and a decreased doubling time compared to when expanded on uncoated TCP (Figure 4A,C).

IA-derived chondrocytes could again be expanded in the Quantum^®^ bioreactor, yielding 68 ± 20 × 10^6^ cells from the 10 × 10^6^ which were seeded over 10.5 ± 0.6 days in culture (Table 3). Similarly to the PD chondrocytes, there was a wide range of Quantum^®^ harvest yields across the different donors, with one donor only yielding 43 × 10^6^ cells and another yielding 90 × 10^6^ cells (Table 3). The % increase in the starting yield was reduced following expansion in the Quantum^®^ compared to TCP with or without vitronectin coating (*p* = 0.02; paired *t*-test; Figure 3B). The total number of population doublings was not significantly reduced following bioreactor expansion compared to TCP (Figure 4B); however, the doubling time was significantly slowed following Quantum^®^ expansion compared to matched TCP expansion (with vitronectin; *p* = 0.01; paired *t*-test; Figure 4D). IA chondrocytes did not demonstrate any differences in total cell yield, number of population doublings or doubling time between TCP coated with or without vitronectin (Figure 3 and Figure 4).

When comparing the potential of the two different donor sources for up-scale expansion, the doubling time in the Quantum^®^ bioreactor was equivalent in chondrocytes from IA (4.4 ± 0.9 days) and PD (3.4 ± 0.8 days) (*p* > 0.05; Mann–Whitney) (Figure 4). Notably, for all the chondrocytes expanded in the Quantum^®^, it was found that multiple harvests with 0.25% trypsin (typically 2–3), incubated for 14 min each, had to be performed in order to harvest all of the cells from the bioreactor. However, even following these multiple harvests and long exposure times to trypsin, the % viability of the cells remained high (>95%). Conversely, chondrocytes could be harvested from the vitronectin-coated TCP flasks using standard 5 min incubation protocols.

### 2.4. Flow Cytometry Markers

**Figure 5:**
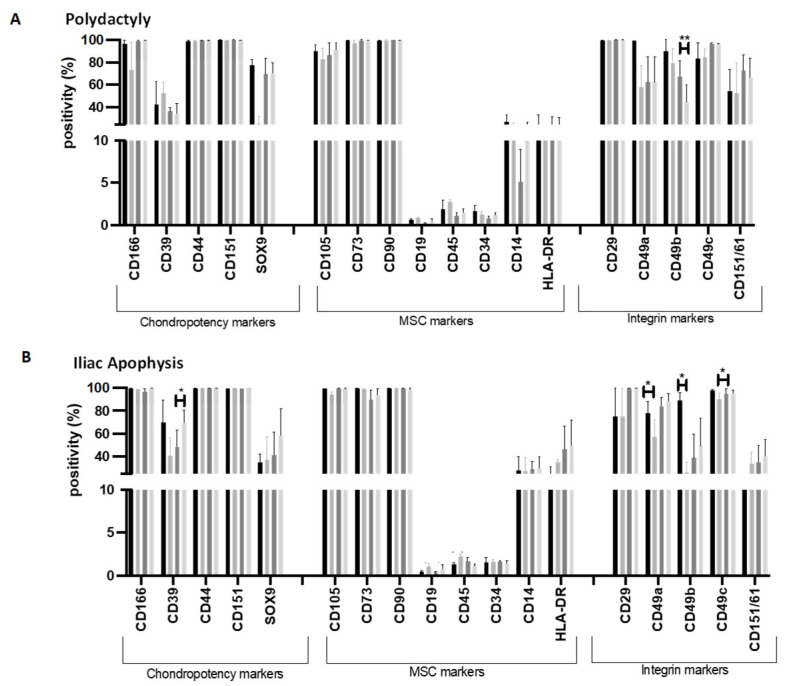
Chondrocyte ((**A**) from polydactyly tissue or (**B**) from iliac apophysis tissue) characterisation using flow cytometry following pre-● and post-Quantum^®^ ●  or matched tissue culture plastic (TCP) expansion with ● or without ● vitronectin coating. (**A**) Polydactyly chondrocytes cultured on plastic coated with vitronectin had higher positivity for CD49b compared to counterparts expanded on uncoated TCP (paired *t*-test; *p* = 0.007). (**B**) Iliac apophysis chondrocytes cultured on plastic coated with vitronectin had decreased CD39 positivity compared to uncoated plastic (*p* = 0.026; paired *t*-test). Quantum^®^ expanded iliac apophysis had increased CD19 positivity compared to matched TCP (with vitronectin) (*p* = 0.044; paired *t*-test). Quantum^®^ expanded iliac apophysis chondrocytes had higher CD45 positivity than pre-Quantum^®^ expanded chondrocytes (*p* = 0.005; paired *t*-test) and reduced CD45 positivity compared to uncoated TCP culture (*p* = 0.013; paired *t*-test). Integrin markers CD49a and CD49b were decreased following Quantum^®^ expansion (*p* = 0.037 and *p* = 0.020, respectively; paired *t*-tests). CD49c was lower following Quantum^®^ expansion compared to matched TCP with vitronectin (*p* = 0.02; paired *t*-test). Data are presented as mean ± the standard deviation of four donor samples per juvenile tissue type. Significance results are represented as * = *p* ≤ 0.05; ** = *p* ≤ 0.01.

PD-derived chondrocytes were negative (<2%) for CD19, CD34 and CD45 and positive (>95%) for MSC markers CD90, CD73 and CD29. Quantum^®^ expansion did not lead to altered expression of chondropotency markers CD166, CD39, CD44, CD151 and SOX9 (paired *t*-test; *p* > 0.05). When comparing between vitronectin-coated and uncoated TCP, CD49b positivity was significantly lower following expansion on uncoated TCP compared to vitronectin-coated TCP (TCP with vitronectin: 67.6 ± 23.8%; uncoated TCP: 44.6 ± 27.0%; paired *t*-test; *p* = 0.007) (Figure 5A).

The IA-derived chondrocytes demonstrated no differences in positivity between Quantum^®^ expanded cells and TCP (vitronectin-coated or uncoated) for MSC markers (CD90, CD73, CD29, CD19, CD34, CD45) or markers related to chondropotency (CD166, CD39, CD44, CD151 or SOX9). Integrin marker, CD49c, however, were significantly decreased following Quantum^®^ expansion compared to matched vitronectin-coated TCP (Quantum^®^: 90.3 ± 9.2%; TCP with vitronectin: 95.1 ± 7.4%; paired *t*-test; *p* = 0.02; Figure 5B). Additionally, Quantum^®^ expansion resulted in decreased positivity of CD49a and CD49b compared to the seeded population ((CD49A: Pre-expansion: 78.0 ± 21.0%; Quantum^®^ expanded: 57.2 ± 30.2%; paired *t*-test; *p* = 0.04) (CD49b: Pre-expansion: 89.2 ± 13.3%; Quantum^®^ expanded: 25.9 ± 18.8%; paired *t*-test; *p* = 0.02) (Figure 5B)).

### 2.5. Gene Expression

**Figure 6:**
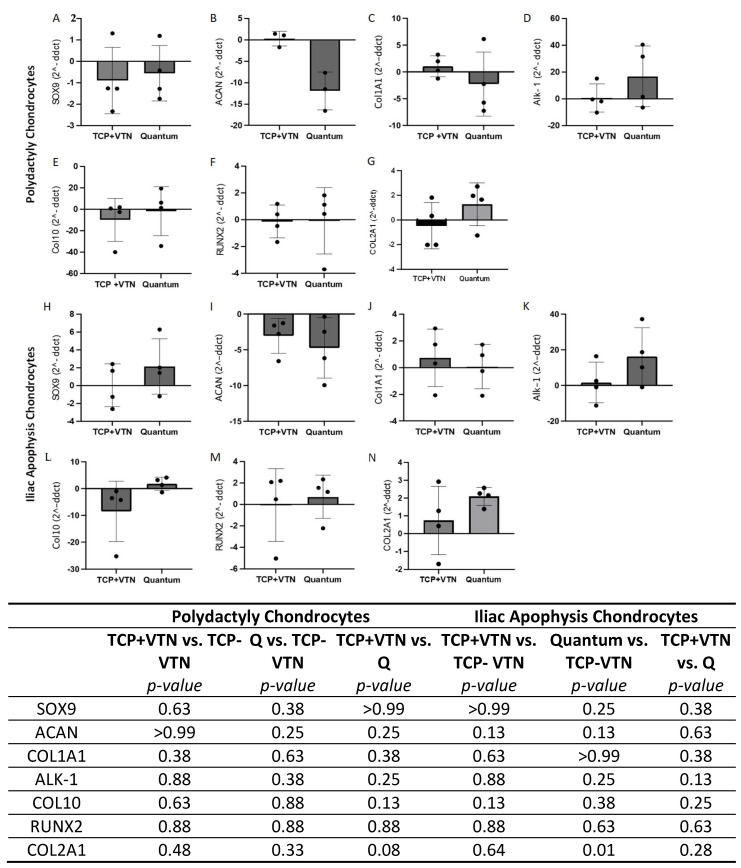
The expression of chondrogenic and hypertrophic genes in chondrocytes from (**A**–**G**) polydactyly and (**H**–**N**) iliac apophysis grown on tissue culture plastic (TCP) coated with vitronectin (+VTN) or in the Quantum^®^ compared to matched donor chondrocytes grown on uncoated TCP. Data are shown as mean ± the standard deviation. No genes demonstrated differential expression following Quantum^®^ expansion compared to matched TCP culture (TCP + VTN) when normalised to uncoated tissue culture plastic (TCP-VTN) (Wilcoxon-matched pairs, *p* > 0.05). Statistical comparisons were also performed to determine whether either Quantum^®^ expansion or vitronectin-coated tissue culture expansion resulted in differential gene expression compared to matched uncoated TCP expansion using Wilcoxon-matched pairs; *p*-value are demonstrated in the table.

The RNA purity was between 1.8 and 2.0 and the RIN was between 9.8 and 10 for all samples. Chondrogenic, hypertrophic and fibrocartilage related gene expression was not altered in either PD or IA chondrocytes between vitronectin-coated TCP expanded and Quantum expanded cells, both normalised to uncoated TCP using the 2-ΔΔCT method (Figure 6; Wilcoxon-matched pairs; *p* > 0.05). Additionally, vitronectin-coated expansion did not result in differential expression of any of the genes compared to uncoated TCP expansion in either chondrocyte type (Figure 6; Wilcoxon-matched pairs; *p* > 0.05). Quantum expanded chondrocytes also retained comparable gene expression to matched cells grown on uncoated TCP (Figure 6; Wilcoxon-matched pairs; *p* > 0.05), with the exception of COL2A1 which had increased expression post-Quantum harvest in comparison to uncoated TCP expansion (Figure 6).

### 2.6. Chrondrogenesis

**Figure 7:**
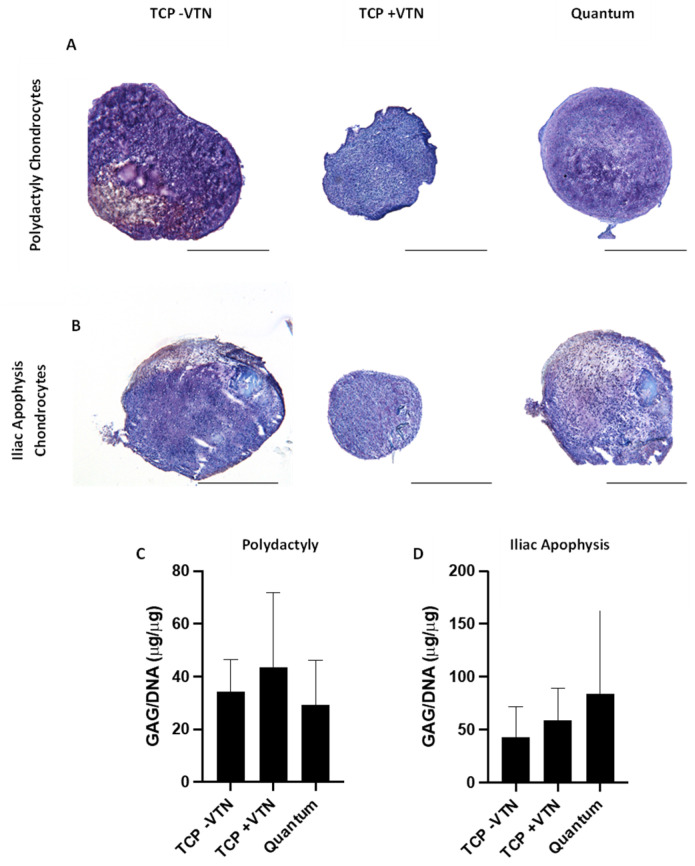
Chondrogenic assessment of pellet cultures from chondrocytes derived from polydactyly and iliac apophysis following expansion in the Quantum^®^ bioreactor or on tissue culture plastic (TCP) coated with vitronectin or uncoated. Representative images of chondrogenic pellets cultured from (**A**) polydactyly-derived and (**B**) iliac apophysis-derived chondrocytes. Chondrogenic pellets were stained using toluidine blue to assess glycosaminoglycans (GAGs). GAGs were measured after chondrogenic differentiation using the DMMB assay and normalised to the DNA content of pellets. Chondrogenic pellets from polydactyly (**C**) and iliac apophysis (**D**) retained equivalent GAG/DNA concentration regardless of culture method (*p* > 0.05; Friedman test). Scale bar represents 500 µm.

Chondrogenic pellets could be established and maintained for 28 days from both PD and IA donors, regardless of the prior culture expansion method. Figure 7 illustrates representative images of chondrogenic pellets stained using toluidine blue to assess GAG content.

Chondrogenic pellets derived from PD and IA chondrocytes had comparable levels of GAG content (normalised to DNA content), regardless of the expansion method (Figure 7; Friedman test; PD *p* = 0.36; IA *p* = 0.65). For PD chondrocyte pellets, the mean GAG/DNA across all sample condition was 35.0 ± 26.5 µg/µg. IA chondrocyte derived comparable GAG content compared to those derived from PD chondrocytes (Mean GAG/DNA across all growth conditions: 62.4 ± 78.8 µg/µg; Mann–Whitney test; *p* = 0.31). The method used to culture expand the IA chondrocytes did not result in any differential production of GAGs in the chondrogenic pellets (Figure 7).

## 3. Discussion

This is the first study, to our knowledge, to expand juvenile donor-derived chondrocytes in a bioreactor for up-scale manufacture. This work demonstrates that both PD and tissue taken from the IA of infants (aged 4 and under) can be used to consistently isolate cells with chondrogenic potential. Both juvenile tissue types could yield equivalent numbers of cells per wet weight of tissue, from which 2D cultures could be established and maintained, retaining their phenotype for multiple passages.

Use of allogenic juvenile cartilage is growing in clinical popularity for the repair of cartilage defects via delivery in a particulated form, also called PJAC [14]. Richards et al. (2023) presented several studies that have demonstrated promising short- to mid-term outcomes following treatment of cartilage defects with PJAC, including a published case study demonstrating well-integrated grafts with no delamination of hypertrophy at 11 years post-treatment [25]. However, other studies have demonstrated graft hypertrophy in 65% of treated individuals (*n* = 20) [26]. The donors from which this product is derived from can be aged up to 13, at which point the regenerative potential of these tissues will start to diminish. The work included in our investigation adds to a growing body of evidence that there may be potential to utilise infant-derived tissues as an attractive alternative. Current therapies derived from infantile PD-derived chondrocytes are being delivered in South Korea and in phase II US trials for treatment of OA [18,19,20,27]. These data provide promising indications that PD chondrocytes may be efficacious and safe for human application. However, the complexity of the existing therapy which requires genetic engineering of the cells to express TGF-β1, may limit the perceived acceptability from patients; gene editing is raising concerns in published patient and public perceptions of cell and gene therapies [28]. Thus, developing expansion and delivery methods for allogeneic PD chondrocytes which are more akin to standard ACI protocols may be perceived as less risky and more attractive to patients.

PD chondrocytes could be expanded in the Quantum^®^ bioreactor, one donor in particular yielding over 110 million cells in a short period of time. Nonetheless, there was considerable donor variability in terms of cell yields; therefore, identification of which donors are likely to produce the greatest yields and retain optimal potency for producing cartilage prior to seeding the Quantum^®^ bioreactor could be beneficial in ensuringmaximal cost-effectiveness through manufacture.

Although up-scale expansion could be accomplished in the bioreactor system, it was notable that the % yield of PD chondrocytes in comparison to the seeded cell number was less and the doubling time was longer in the Quantum^®^ compared to the matched culture on TCP. This phenomenon was also identified when IA-derived chondrocytes were cultured in the bioreactor system. Previous attempts to expand adult chondrocytes in this system also resulted in a trend towards slower doubling time and only a comparable % increase in starting yield compared to TCP-matched culture [12]. For the vast majority of other cell types, Quantum^®^ expansion results in significantly higher % cell yields compared to standard culture [13,29]. Together, these studies indicate that further optimisation of the bioreactor expansion process may be required for chondrocyte manufacture. Currently, many of the protocols developed with the support of the supplier, TerumoBCT, are focused upon expansion of MSCs, around which the flow rates, adhesion times and processes have been developed [13]. Future work is required to optimise every step of the expansion process, to determine whether chondrocyte expansion in the Quantum^®^ can be made more efficient. This includes identification of optimal GMP-compliant serum-free media, collagenases and vitronectin, as well as determining exact adhesion times for the cells onto vitronectin-coated polysulfone fibres (which make up the bioreactor) and further assessment of the metabolic rate of these cells to ensure lactate values can be used as an accurate proxy of cell growth to maintain them in an active growth phase. Alternatively, it may be that other GMP-compliant bioreactors may be better suited for the purposes of up-scale GMP chondrocyte manufacture, which should be tested in future studies.

Regardless of the limitations in terms of slowed doubling time in the Quantum^®^, it was reassuring that the cells that were yielded from both juvenile donor sources were comparable to matched cells grown on TCP in terms of their chondrogenic potential. IA chondrocytes retained equivalent expression of cell surface markers relating to chondrogenic potency and stemness. Notably, CD49c (Integrin alpha 3) was decreased following Quantum^®^ expansion compared to matched coated TCP expansion. Additionally, these chondrocytes maintained comparable expression of chondrogenic, hypertrophic and fibrocartilage related genes, produced equivalent amounts of GAGs normalised to DNA content, and could produce chondrogenic pellets. It would have been interesting to plastic culture the Quantum^®^-expanded chondrocytes to determine whether their growth rates could be increased or whether the growth phenotype of the cells was irreversibly altered. Thus, it suggests that Quantum^®^ expansion does not lead to de-differentiation of these cells and even if the cell yields are not as expected, this GMP-compliant method should still be considered as it requires much lower levels of manual handling and can be used in a class C or D clean room, as opposed to ‘open-manufacture’ using TCP which would require access to a class A clean room.

PD chondrocytes retained equivalent gene expression, GAG/DNA concentrations and could produce chondrogenic pellets regardless of their expansion methods. However, SOX9 surface marker expression was reduced in Quantum^®^ expanded chondrocytes compared to their pre-expansion levels, perhaps indicating some level of de-differentiation post-Quantum^®^ expansion. Further work is required to determine the consequences of this observation, particularly when measurement of SOX9 was not altered compared to TCP expanded cells at the same passage and as the SOX9 gene in matched cells did not demonstrate different expression levels. Measurement of the SOX9 transcription factor is widely regarded as a marker of chondrocyte differentiation and proliferation, being recognised as a key driver of chondrocyte potency and a master regulator of chondrocyte function [30]. Furthermore, although no significant difference in GAG/DNA concentrations of chondrogenic pellets was demonstrated, there was notable variation in GAG/DNA concentrations per pellet within donor and across donors. Variation in GAG/DNA readouts from chondrogenic pellets assays have been identified widely and highlight the need for optimal, high-throughput assays to determine chondrogenic function [31].

PD and IA both offer attractive alternative donor sources for deriving allogeneic chondrocytes. To allow further translation of this work towards a clinical product, careful development of GMP-compliant protocols would be required. This includes the establishment of rigorous release criteria including sterility, viability, identity, stability/functionality indicators and thresholds and potentially genetic screening. Significant additional safety testing of the PD-derived chondrocytes would be required as there are several known causes of polydactyly development, including genetic malformations and conditions such as ciliopathies [32]. Scrutiny of the potential effects of the different polydactyly causes on recipients of the cells would require assessment through a series of preclinical studies in which donor variability can be assessed. Furthermore, potency criteria for the allogenic cells will be required as part of a product release criteria for any future ATMP delivered in Phase II-III trials [33].

## 4. Materials and Methods

### 4.1. Patients

Samples were collected with parental informed consent from polydactyly patients undergoing surgery to remove additional digits. Small samples (≈1 g) of IA were collected during open reduction or Salter’s osteotomy surgeries for the treatment of hip dysplasia, following informed parental consent. Ethical approvals were obtained from the National Research Ethics Service- North West Committee (17/NW/0550; approval date: 19 October 2017).

### 4.2. Chondrocyte Isolation and Expansion

PDs were carefully dissected, under sterile conditions, to isolate joints. Sagittal slices through the joint were dissected and retained for histological analysis. The cartilage-like tissue on either side of the joint was dissected, imaged, weighed and minced for digestion. Likewise for the IA tissue, a slice was dissected under sterile conditions and retained for histological analysis. The remaining IA tissue was imaged, weighed and processed for digestion. Cartilage from both donor sources was minced into small pieces (1 mm^3^) and then digested, on a roller, overnight at 37 °C for 16 h. Collagenase type II (250 IU/mg dry weight, Worthington, NJ, USA) in serum-free Dulbecco’s Modified Eagle’s Medium/F-12 (DMEM/F-12; Life Technologies, Paisley, UK) was used for the digestion. The following morning, a 40 µm cell strainer was used to strain the media/cell suspension. The cells were then pelleted by centrifugation at 350× g for 10 min. Cells were counted and viability assessed using trypan blue exclusion. Cells were seeded onto tissue culture flasks (Sarstedt, Leicester, UK) at a density of 5 × 103 cells/cm^2^ in DMEM-F12 with 1% (*v*/*v*) penicillin/streptomycin (P/S; Life Technologies, Paisley, UK), 10% foetal bovine serum (FBS; Life Technologies, Paisley, UK) and 62 μg/mL ascorbic acid (VWR, Radnor, PA, USA). Chondrocytes were maintained in a humidified atmosphere at 37 °C with 21% O_2_ and 5% CO_2_ for 4–6 days, after which the media was replenished. Chondrocytes were then fed every 2–3 days until they reached 70–80% confluency. This culture expansion phase is referred to as P0.

Once 70–80% confluency was reached, chondrocytes were removed from the plastic using trypsin and then re-seeded at 5 × 10^3^ cells/cm^2^ until sufficient cells yields were achieved such as to seed the Quantum^®^ bioreactor and perform parallel tissue culture plastic (TCP) experiments.

### 4.3. Histological Analysis of the Juvenile Cartilage Tissues

Longitudinal slices through the PD joints and full depth IA tissue sections were fixed in 10% neutral buffered formalin prior to embedding in paraffin wax. Five-micrometre sections of tissue were collected onto Polysine^®^ slides (ThermoScientific, Braunschweig, Germany) and stained with either toluidine blue to assess proteoglycan content or haemotoxylin and eosin (H&E) to assess general morphology [34] prior to assessment via bright-field microscopy. The collagen fibre organisation and orientation were assessed microscopically using polarised light.

### 4.4. Up-Scale Chondrocyte Manufacture in the Quantum^®^ Cell Expansion System

The Quantum^®^ cell expansion system was set-up, using a new, sterile research use only (RUO) bioreactor for each donor’s expansion. The bioreactor was coated overnight with 4 mg of truncated, recombinant vitronectin (Gibco, Life Technologies, Paisley, UK) to allow for adherence of cells to the polysulphone hollow fibres. Vitronectin was selected as the coating source, as GMP-compliant counterparts are commercially available, allowing easy transfer for clinical application. Vitronectin and/or fibronectin provide more reproducible coating and are easier to obtain than GMP-compliant pooled human cryoprecipitate, as used in our previous studies [12,29]. The bioreactor system was then conditioned for at least 1 h with chondrocyte media (DMEM-F12 with 1% (*v*/*v*) P/S; (Life Technologies, Paisley, UK), 10% FBS (Life Technologies, Paisley, UK) and 62 μg/mL ascorbic acid (VWR, Radnor, PA, USA)). A total of 10 × 10^6^ chondrocytes were then seeded into the Quantum^®^ cell expansion system and allowed to adhere for 24 h with uniform suspension. Chondrocyte media was continuously perfused through the Quantum^®^ cell expansion system, removing an equal volume of conditioned medium. Cellular metabolism was assessed throughout the culture period as a proxy of chondrocyte proliferation. Metabolism was assessed through measuring concentrations of lactate and glucose in the conditioned medium using a Lactate Plus metre (Nova Biomedical, Runcorn, UK) and a clinical blood glucose metre (Kinetik Wellbeing, Redhill, UK), respectively. The culture medium perfusion rate was increased as the number of cells expanded, from a baseline rate of 0.1 mL/min to 0.8 mL/min. Cell growth (via lactate and glucose measurement) was plotted daily to ensure cells were maintained in an expansion phase. Once the cell growth started to plateau, chondrocytes were harvested.

### 4.5. Chondrocyte Parallel ‘Sister’ TCP Culture

Table 2 and Table 3 detail the passage at which chondrocytes were seeded into the Quantum^®^ or onto parallel TCP cultures. The day preceding the set-up of ‘sister’ TCP flasks, T175 tissue culture vessels (Sarstedt, Leicester, UK) were coated with 440 µg vitronectin (same batch used for Quantum^®^ coating of matched cultures). Parallel ‘sister’ TCP cultures were seeded onto plastic at 500 cells/cm^2^ to mirror the seeding density in the Quantum^®^ bioreactor. Chondrocytes were seeded onto TCP flasks coated with vitronectin or without any addition of a substrate (uncoated) to replicate our standard laboratory practice [35].

### 4.6. Calculation of Growth Kinetics

Cell counts were performed at the time of trypsinisation. The number of population doublings was calculated using the following formula: $PD\;=\;3.32\;*\;\left(logN_{2}\;-\;logN_{1}\right)$, where ***N1*** is the cell number seeded and ***N2*** is the harvest cell number. The following formula was used to calculate the chondrocyte doubling time ***(DT):*** $DT\;=\;\left(t2\;-\;t1\right)\;*\;(\frac{\ln\left(2\right)}{\ln\left(\frac{n2}{n1}\right)})$, where ***t1*** is the time at seeding, ***t2*** is the time at harvesting, ***n1*** is the cell number at seeding and **n2** is the cell number at harvest.

### 4.7. Flow Cytometry Profiling

Chondrocytes were harvested and profiled by flow cytometry immediately prior to seeding the Quantum^®^ or TCP sister flasks, as well as on the day of their harvest. Cells were harvested by trypsinisation, centrifuged, counted and 2 × 10^4^ cells added per tube. Chondrocytes were blocked in 10% human IgG made in 2% bovine serum albumin (BSA) for 30 min and then centrifuged at 500× g for 5 min, prior to resuspension in 2% BSA. A panel of antibody markers were analysed to assess MSC profiles [36], chondrogenic potency [37] and integrins. For all antibodies, isotype-matched IgG controls were used for gating (all Becton Dickinson and Company, Oxford, UK). The fluorochrome-conjugated antibodies (all Becton Dickinson and Company, Oxford, UK) used to assessed MSC profiles were CD73-BV421 (clone AD2), CD90-Phycoerythrin (PE) (clone 5E10), CD105-APC (clone 266), CD45-PE (clone HI30), CD14-PerCP-Cy5.5 (clone MϕP9), CD19-BV421 (clone HIB19) and CD34-APC (clone 581). For assessing chondrogenic potency, the antibodies used were CD44-Peridinin-chlorophyll proteins-Cyanine 5.5 (PerCP-Cy5.5) (clone G44–26), CD166-Brilliant Violet 421 (BV421) (clone 3A6), CD39-Allophycocyanin (APC) (clone TU66) and CD151-PE (clone14A2.H1). Antibodies used for the assessment of integrin profiles were CD151/61-PE (clone 23C6), CD29-APC (MAR4), CD49b-BV421 (clone 12F1), CD49a-PE (clone SR84) and CD49c-PE (clone C3 II.1). Intracellular SOX-9 (clone 3C 10; Abcam, Cambridge, UK) was also measured as a marker of chondrogenic potency. To measure intracellular SOX-9, cells were fixed with 80% (*v*/*v*) methanol, permeabilised in 0.1% (*v*/*v*) tween-20 in phosphate-buffered saline (PBS) before blocking in 10% human IgG made in 0.1% (*v*/*v*) tween-20 in PBS. Finally, cells were resuspended in 0.1% (*v*/*v*) tween-20 in PBS for flow cytometry. A FACSCanto II flow cytometer was used with Diva 7 software (Becton Dickinson and Company, Oxford, UK).

### 4.8. Gene Expression Analysis Using RT-qPCR

RNA was extracted from both PD and IA samples following expansion on uncoated and vitronectin-coated tissue culture plastic (TCP) or in the Quantum^®^ bioreactor coated with vitronectin. Extraction was performed using the RNeasy mini kit (Qiagen, Manchester, UK) according to the manufacturer’s instructions. Two 25 µL aliquots of RNA was stored in the −80 °C freezer until needed and the remainder was used for quality and quantity checks of the extracted RNA.

To assess the RNA concentrations and purity, 2 µL of each RNA sample was added to a LVis plate and the plate was read using a FLUOStar Omega microplate reader (BMG Labtech, Ortenberg, Germany). Purity was determined by A260/A280 ratio [38]. The quality of the extracted RNA was assessed using the TapeStation 4150 (Agilent, Stockport, UK) to determine the RNA integrity number (RIN).

Following the RNA extraction and carrying out quality and quantity checks, 1000 ng RNA of each sample was converted to cDNA using a high-capacity cDNA reverse transcription kit (Applied Biosystems™). RT-qPCR analysis was performed using SYBR green reagent (Thermo Fisher Scientific, Oxford, UK) and QuantiTect Primer Assays (Qiagen, Manchester, UK) to assess markers of chondrogenic potency: collagen I (COL1A1), collagen II (COL2A1), SOX9 and aggrecan (ACAN). Additionally, the expression of de-differentiation-related genes, activin receptor-like kinase-1 (ALK-1) and collagen X (COL10A1), and osteoblast differentiation-related gene, Runt-related transcription factor 2 (RUNX2) was assessed. Glyceraldehyde-3-phosphate dehydrogenase (GAPDH), hypoxanthine phosphoribosyltransferase 1 (HPRT1) and TATA box-binding protein (TBP) were used as reference genes. All RT-qPCR was performed on the QuantStudio™ 3 RT-qPCR system (Applied Biosystems, Waltham, MA, USA). The Ct values were determined using the QuantStudio software (v1.3.1) (Applied Biosystems, Waltham, MA, USA); the expression of genes of interest in the experimental conditions were normalised to the reference genes and expressed relative to the control (TCP without vitronectin) using the 2-ΔΔCT method [39]. A 2-fold change in expression was considered biologically significant.

### 4.9. Chondrogenic Pellet Culture

Following the harvest of chondrocytes from the Quantum^®^ or sister TCP flasks, chondrogenic pellet cultures were established. Chondrogenic differentiation media was made comprising DMEM-F12 (Life Technologies, Paisley, UK) containing 10 ngml^−1^ Transforming Growth Factor- β (TGF-β; PeproTech, Rocky Hill, NJ, USA), 1 mM sodium pyruvate, 10 μM dexamethasone, 1 mM ascorbic acid-2-phosphate and 20 μM linoleic acid (all Sigma Aldrich, Gillingham, UK), 1% Insulin Transferrin Selenium (ITS-G; GibcoTM, Fisher Scientific, Paisley, UK) and 1% P/S (Life Technologies, Paisley, UK). Pellets of 2 × 10^5^ chondrocytes were centrifuged at 500× g for 8 min in chondrogenic differentiation media. Chondrogenic pellets were then maintained at 37 °C with 21% O_2_ and 5% CO_2_. After three days of static culture, the pellets were dislodged from the Eppendorf tube, following which chondrogenic pellet media was changed every 2–3 days. After 28 days in culture, the pellets were washed with PBS (Life Technologies, Paisley, UK), snap-frozen in liquid nitrogen then stored at −80 °C until subsequent analysis.

### 4.10. Analysis of Chondrogenic Pellet Glycosaminoglycan (GAG)/DNA Content

A papain solution (125 µg/mL) was made up in a buffer of 5 mM EDTA, 5 mM cysteine hydrochloride and 0.1 M sodium phosphate (all Sigma Aldrich, UK), adjusted to pH 6, was used to digest the chondrogenic pellets. Pellets in buffered papain were digested at 60 °C for 3 h, with vortexing being performed every 30 min, before being centrifuged at 1000× g for 5 min and the supernatant stored at −20 °C for subsequent analysis.

A dimethylmethylene blue (DMMB) assay was used to quantitate GAGs [39,40]. Chondroitin sulphate from bovine trachea (Sigma Aldrich, UK) was used to prepare a serial dilution of standards from 0 to 20 µg/mL in PBS. An amount of 200 µL of 4× dmmb staining solution and 50 µL of sample or standard were added to wells of a 96-well plate and the absorbance read immediately at 530 nm. A standard curve was used to calculate the GAG content for each sample. The amount of double stranded DNA in each pellet was quantitated using a picogreen assay (Invitrogen, Waltham, MA, USA), performed according to the manufacturer’s instructions. Fluorescence was measured on a plate reader (Omega FLUOStar, BMG Labtech, Ortenberg, Germany) with excitation at 480 nm and emission at 520 nm. For each chondrogenic pellet, the GAG content was divided by its DNA content to calculate the concentration of GAGs per concentration of DNA.

### 4.11. Analysis of Chondrogenic Pellets by Histology

Chondrogenic pellets were snap-frozen on filter paper in liquid nitrogen-cooled hexane, then cryosectioned at 7 µm thick onto Polysine^®^ slides (ThermoScientific, Braunschweig, Germany). Sections were stained with toluidine blue to assess proteoglycan content via bright-field microscopy.

### 4.12. Statistical Analysis

Prism software version 9.0 (GraphPad Software, San Diego, CA, USA) was used to perform statistical analysis. The normality of the data was assessed using a Shapiro–Wilk test, which was used to inform whether parametric or non-parametric statistical tests were appropriate. Unpaired data were analysed using an unpaired Student *t*-test or Mann–Whitney U test, where appropriate, based on the normality of the dataset. Paired data were analysed using a paired *t*-test or a Wilcoxon-matched pairs signed rank test, where appropriate. For multiple comparisons, analysis of variance (ANOVA) or Kruskal–Wallis were used, with either a Holm–Sidaks or Dunn’s multiple comparisons post hoc test, respectively, where appropriate. *p* values ≤ 0.05 were considered significant.

## Figures and Tables

**Table 1:** Demographics of patients undergoing surgery for the removal of polydactyly digits or undergoing surgical procedures (Salter’s Osteotomy or Open Reduction) for the treatment of juvenile hip dysplasia from which cartilaginous tissues were harvested.

Sample Type	Side	Donor	Gender	Age (Years, Months)
Polydactyly Digit	Right	1	F	1 y 2 mo
Polydactyly Digit		2	M	0 y 9 mo
Polydactyly Digit	Right	3	M	3 y 2 mo
Polydactyly Digit	Left	4	M	1 y 9 mo
Iliac Apophysis	Right	1	F	2 y 0 mo
Iliac Apophysis	Left	2	F	2 y 2 mo
Iliac Apophysis	Right	3	F	1 y 3 mo
Iliac Apophysis	Left	4	F	2 y 0 mo

**Table 2:** Cell growth characteristics of polydactyly-derived chondrocytes in the Quantum^®^ and on tissue culture plastic (TCP) with or without vitronectin coating.

	Donor	1	2	3	4	Mean	SD
Tissue digested (g)		0.23	0.069	0.041	0.015	0.089	0.097
Cell yield (×10^6^)		0.62	0.17	0.11	0.25	0.29	0.23
Passage for Quantum^®^ and matched TCP expansion		1	2	6	3	3	2.2
TCP vitronectin coated	Cells seeded (×10^6^)	0.875	0.875	0.875	0.875	0.875	0.0
	Cells harvested (×10^6^)	11.78	4.17	9.10	11.01	9.02	3.4
	Increased cells Yield (×10^6^)	10.9	3.3	8.2	10.1	8.13	3.4
	Days in culture	9	7	10	11	9.3	1.7
	Doubling time (days)	1.3	1.3	1.5	1.6	1.4	0.2
	Population doublings	7.1	5.6	6.7	7.0	6.6	0.7
TCP-not vitronectin coated	Cells seeded (×10^6^)	0.875	0.875	0.875	0.875	0.875	0.0
	Cells harvested (×10^6^)	8.01	2.74	6.24	7.04	6.01	2.3
	Increased cells Yield (×10^6^)	7.1	1.87	5.37	6.17	5.13	2.3
	Days in culture	9	7	10	11	9.25	1.7
	Doubling time (days)	1.4	1.4	1.6	1.7	1.5	0.2
	Population doublings	6.5	5.0	6.2	6.3	6.0	0.7
Quantum^®^- vitronectin coated	Cells seeded (×10^6^)	10	10	10	10	10	0.0
	Cells harvested (×10^6^)	96	53	107	110.8	91.7	26.6
	Increased cells Yield (×10^6^)	86	43	97	100.8	81.7	26.6
	Days in culture	10	11	10	11	10.5	0.6
	Doubling time (days)	3.1	4.6	2.9	3.2	3.4	0.8
	Population doublings	3.3	2.4	3.4	3.5	3.1	0.5

**Table 3:** Cell growth characteristics of iliac apophysis-derived chondrocytes in the Quantum^®^ and on tissue culture plastic (TCP) with or without vitronectin coating.

	Donor	1	2	3	4	Mean	SD
Tissue digested (g)		0.140	0.117	0.748	0.138	0.286	0.31
Cell yield (×10^6^)		1.05	1.17	0.99	0.625	0.959	0.23
Passage for Quantum^®^ and matched TCP expansion		3	3	3	3	3	0.0
TCP vitronectin coated	Cells seeded (×10^6^)	0.875	0.875	0.875	0.875	0.875	0.0
	Cells harvested (×10^6^)	6.02	6.15	4.32	2.95	4.86	1.5
	Increased cells Yield (×10^6^)	5.15	5.28	3.45	2.08	3.99	1.5
	Days in culture	11	11	13	11	11.5	1.0
	Doubling time (days)	1.8	1.8	2.3	2.2	2.0	0.3
	Population doublings	6.1	6.1	5.6	5.1	5.7	0.5
TCP-not vitronectin coated	Cells seeded (×10^6^)	0.875	0.875	0.875	0.875	0.875	0
	Cells harvested (×10^6^)	4.73	3.98	4.83	1.61	3.79	1.5
	Increased cells Yield (×10^6^)	3.86	3.1	4.0	0.7	3.7	1.5
	Days in culture	11	11	13	11	11.5	1.0
	Doubling time (days)	1.9	2.0	2.3	2.6	2.2	0.3
	Population doublings	5.8	5.5	5.8	4.2	5.3	0.8
Quantum^®^- vitronectin coated	Cells seeded (×10^6^)	10	10	10	10	10	0
	Cells harvested (×10^6^)	62	90	76	43	67.8	20.1
	Increased cells Yield (×10^6^)	52	80	66	33	57.8	20.1
	Days in culture	11	12	11	12	11.5	0.6
	Doubling time (days)	4.2	3.8	3.8	5.7	4.4	0.9
	Population doublings	2.6	3.2	2.9	2.1	2.7	0.5

## Data Availability

Data will be made available upon request to the corresponding author.
